# Supplementary material for: Are carbon emissions trading and green financial instruments synergistic? -Comprehensive quantitative research based on content analysis
Source: PLoS One. 2024 Mar 7;19(3):e0298601. doi: 10.1371/journal.pone.0298601 (PMC10919601; doi:10.1371/journal.pone.0298601)
Supplement: S1 Data — (ZIP) [file pone.0298601.s001.zip › Data/地级市Z_F工作报告.zip!/╣π╢1⁄2/│▒╓▌╩╨/└┤╫╘╣1⁄2╓┌║┼í╛┬φ┐╦╩2╛▌═°í┐_2005.docx]

各位代表：

我代表市人民政府向大会作工作报告，请予审议，并请政协各位委员和其他列席人员提出意见。

2004年的工作回顾

过去的一年，在省委、省政府和市委的正确领导下，在市人民代表大会及其常委会和市政协的监督支持下，市政府团结和带领全市人民，以邓小平理论和“三个代表”重要思想为指导，深入贯彻党的十六大及十六届三中、四中全会，省委九届四次、五次全会和市委十一届四次、五次全体会议精神，牢固树立和认真落实科学发展观，抓好发展这个第一要务，按照建设“活力潮州、文化潮州、生态潮州、法治潮州”的总体思路，进一步解放思想，坚定信心，发挥优势，抢抓机遇，务实进取，使国民经济保持持续较快发展，社会事业全面进步，精神文明和民主法制建设取得新进展，城市活力进一步增强，魅力进一步展现，较好地完成市十二届人大二次会议确定的各项任务。

（一）国民经济持续较快发展，经济实力明显增强

人均生产总值突破万元。全市实现生产总值256.9亿元，比上年增长11.1%。其中，第一产业增加值34.14亿元，增长1%；第二产业增加值125.25亿元，增长15.4%；第三产业增加值97.53亿元，增长9.2%。三次产业结构为13.3 :48.8 :37.9。全市人均生产总值10306元，增长6.1%。

财税收入增幅较大。全市财政总收入20.36亿元。地方财政一般预算收入5.25亿元，增长17.6%（老口径7.12亿元，增长20.8%）。各项税收总额20.4亿元，增长28.4%。其中，国税税收13.5亿元，增长30.9%，地税税收6.9亿元，增长23.7%。

金融运行平稳。去年底，全市金融机构本外币存款余额281.83亿元，比年初增长17.8%。其中，居民储蓄存款余额212.49亿元，增长17.9%。各项贷款余额132.68亿元，增长7.3%。

（二）农村经济稳步发展，县域经济实现新突破

特色农业发展加快。认真落实扶持粮食生产的各项政策，狠抓粮食生产。全年粮食播种面积103.18万亩，增加5.1万亩，总产量45.06万吨，增加2.36万吨，全面完成省下达的考评指标。大力发展茶叶、生柑、花卉和优质水产品等特色农业，生产基地规模逐步扩大，种养品种结构不断优化。农业新技术进一步推广，农业标准化和农产品质量安全工作得到加强。

­农业产业化积极推进。继续扶持发展农业龙头企业，国家、省、市认定的农业龙头企业达22家，带动农户5.9万户，户均增收1200多元。大力培养农产品流通大户，列为扶持对象的7家流通大户年购销农产品3.84万吨，销售收入2.54亿元。组织农业企业参加广东省农副产品（成都）交易会、第二届中国国际农产品交易会和中国国际茶业博览会，取得明显成效。

抗击禽流感取得阶段性胜利。潮安县古巷镇发生高致病性禽流感疫情后，全市各级积极行动，采取果断措施，及时对疫区家禽进行扑杀，对全市禽类实施强制免疫，有效控制了疫情，受到省委、省政府表彰。

农村政策进一步落实。农村税费改革成果得到巩固，农民负担由税费改革前的人均109.61元减至4.54元，减负达到95.9%。积极推进二轮土地承包工作。农村财务管理和审计工作得到加强，会计委派（选聘）和代理制试点工作取得初步成效。山区扶贫工作扎实开展，农村劳务经济进一步发展。

县域经济发展加快。认真落实省加快县域经济发展的决定及相关政策措施，大力发展特色经济，加大招商引资力度，促进工业增效，农民增收，财政增强。潮安县实现生产总值105.23亿元，增长12.5%；地方财政一般预算收入1.34亿元，增长26.3%（老口径1.63亿元，增长28.1%）。饶平县实现生产总值71.39亿元，增长8.2%；地方财政一般预算收入0.62亿元，增长1.1%（老口径0.83亿元，增长8.4%）。

（三）特色工业显现活力，民营经济不断发展壮大

工业运行质量明显改善。全市工业总产值467亿元，其中规模以上工业总产值206亿元，增长27.7%，增速居全省各市第四位；实现工业销售产值204.58亿元，增长30.1%；实现利税10.14亿元，增长24.5%。工业经济效益综合指数111.15%，提高10.8个百分点。燃气生产和供应发展迅猛，增长66.6%；工业供电量14.8亿千瓦时，增长25.2%。

支柱行业主导作用明显。规模以上七大行业实现增加值31.4亿元，增长25%，占规模以上工业增加值的65%。陶瓷工业支柱作用突出，实现增加值13.1亿元，增长37%，拉动规模以上工业增幅上升9.8个百分点。

品牌带动战略成效突出。争创区域品牌取得丰硕成果。先后获得“中国优秀旅游城市”、“中国婚纱晚礼服名城”、“中国瓷都”、“中国潮州菜之乡”等称号，潮安县彩塘镇获得“中国不锈钢制品之乡”称号。争创国家名牌产品、国家驰名商标实现零的突破。笑咪咪糖果、顺发不锈钢压力锅被评为中国名牌产品，雅士利商标被评为中国驰名商标。还有3个产品获国家免检产品称号，15个产品获省名牌产品，18个商标获省著名商标。企业获得省级以上品牌累计达74个。

企业创新能力进一步增强。技术改造步伐加快，完成技改投资12.6亿元，增长24.4%。民营科技企业进一步壮大，被省认定为民营科技企业达134家，被省认定为高新技术企业达60家。全市高新技术产品产值63亿元，增长65%。以企业为主体的科技创新体系进一步完善，市级工程技术中心已有16家，省级工程技术中心7家，省级农业科技创新中心2家。在省科技厅对各市进行的区域创新能力评估中，我市企业创新能力名列第8位。科技计划项目成果显著，有5个项目列入国家项目计划，19个项目列入省技术改造重点项目计划。三环集团“BMB高积层片式陶瓷电容器”、华德公司“日用高温红陶产品开发”等技术创新项目分别达到国际、国内先进水平。

（四）文化旅游发展加快，第三产业发展水平提高

“文化潮州”建设取得新进展。成立潮州文化研究中心，开展“潮州韩愈现象”研究。与中山大学建立了“共建文化潮州、营造和谐社会”市校合作关系。成功举办首届潮州文化旅游节和《魅力潮州》电视艺术采风活动。组队参加第五届全国烹饪技术比赛获得团体金奖。加快文化设施建设，市文化艺术馆、图书馆新馆、博物馆、档案馆、饶宗颐学术馆、太平路牌坊街等重点文化设施前期工作扎实推进。加强重点文物古迹保护，广济桥维修一期工程进展顺利，二期工程正在进行施工设计；许驸马府维修已完成土木工程；从熙公祠、韩文公祠维修工程全部完成。重视挖掘保护民间文化遗产，潮州民间工艺美术陈列馆、广济桥史陈列馆、潮州戏曲史陈列馆筹建工作积极进行。人民广场、滨江长廊和社区的文化活动日益丰富。一批文艺作品在省、国家及国际上获奖。重视发展新兴文化产业，积极扶持“宇航鼠”动漫产业项目。网吧管理及音像市场专项整治取得明显成效。

旅游经济持续升温。旅游环境进一步完善，旅游设施进一步配套，旅游服务水平进一步提高。旅游区域结构得到有效调整，生态特色旅游初具规模。凤凰山生态旅游区、星湖温泉度假村等项目进展顺利。旅游扶贫工作成效明显，道韵楼、龙湖寨、绿岛山庄、中华名茶园、白水岩风景区5个项目被列入省旅游扶贫项目。由宽带网支撑的潮州市旅游信息咨询系统在省内率先投入使用。旅游区域合作不断扩大，开辟闽粤赣十三市无障碍旅游区和闽粤旅游大走廊。全年共接待海内外游客143.35万人，增长29%；旅游收入20.74亿元，增长28%。

市场消费明显趋旺。汽车、通讯器材、商品房等大宗商品消费保持较快增长，其中，新购置入户的小汽车6654辆，增长18.9%。连锁经营、物流配送等现代流通业发展态势良好。广告、医疗保健、餐饮、保险等服务业稳步发展。全年社会消费品零售总额91.2亿元，增长11.5%。

（五）开拓市场成效显著，外源型经济发展上新台阶

市场多元化战略取得明显成效。在北京成功举办了“中国瓷都——广东潮州陶瓷博览会”；在广东国际服装博览会上，成功举办了“中国婚纱晚礼服杯”国际服装名校学生作品大赛和“潮州婚纱晚礼服之夜”主题晚会。认真组织企业参加两届广交会、中小企业博览会和泛珠三角区域经贸合作洽谈会、第三届“山洽会”等国家和省举办的经贸展销洽谈活动，充分展示我市的特色产业优势，提高了潮州产品的市场占有率。

对外贸易稳步增长。全年外贸进出口总值15.34亿美元，增长31.8%。其中出口11.79亿美元，增长25.4%，首次突破十亿美元大关。出口总值中，一般贸易出口10.34亿美元，增长27.1%；加工贸易出口1.44亿美元，增长14.4%。陶瓷、服装是我市出口较快增长的支柱，占出口总额的65.5%，其中陶瓷出口5.6亿美元，增长37.1%，所占比重为47%。高新技术产品和机电产品出口增幅较大，分别增长80.4%和50.1%。

招商引资有新突破。新批外商投资项目58个，比上年增加15个。合同利用外资1.01亿美元，增长1.5倍。发达国家投资不断增加，来自日本、韩国、英国、法国、美国、澳大利亚等国家的项目16个，合同利用外资5671万美元。全市实际利用外资总额0.6亿美元，增长36.8%。利用市外境内资金达23.03亿元，也上了一个新台阶。

（六）固定资产投资力度加大，基础设施建设取得新突破

投资增速创历史新高。多渠道筹集资金，增加固定资产投资，拉动经济增长，增强了城市吸引力和竞争力。全社会固定资产投资总额75.42亿元，增长57.5%。城镇50万元以上项目投资额68.25亿元，增长72.5%。投资结构进一步优化，基本建设投资41亿元，增长1.4倍；更新改造投资15.3亿元，增长23%；房地产开发投资3.5亿元，下降8%。

基础设施不断完善。30个在建重点项目进展顺利，累计完成投资28.02亿元。潮州供水枢纽、三百门电厂首期工程、省道丰柏线潮州段改造工程、饶平沿海片改水工程、城乡电网改造和输变电工程等项目进展顺利；潮揭高速公路、韩江北桥、市垃圾卫生填埋场、福利院、全民健身广场、货柜车查验场、开发区二期工业园区、韩江南堤首段整治工程等项目动工建设；第一污水处理厂扩建工程、潮汕公路拓宽改造工程、凤凰水电厂改造扩容工程、饶平县高沙水闸等投入使用；县通镇、镇通村公路硬底化建设加快；厦深高速铁路已列入铁道部规划，正式开展设计招投标工作。

城市建设上新台阶。创建“国家园林城市”活动扎实推进，城市净化、绿化、美化、亮化的水平不断提高。人民广场和文化公园建成开放，潮州大道、汕汾高速公路引道完成改造和绿化工作，韩江东岸、北关引韩整治工程已经竣工，城市环境有了较大改善。推行专业化、市场化的模式，提高了城市环境卫生、绿化养护的管理水平。枫春路、城新路、新洋路创建文明样板路试点收到良好成效。落实城市管理执法责任制，加强对市区“脏乱差”问题的整治，市容更加整洁有序。

（七）各项社会事业不断进步，可持续发展能力逐步增强

科技工作进一步加强。市政府与8个高等院校、科研机构建立了合作关系。多渠道筹集资金2000多万元，建立了市科技创新服务中心。组织实施国家和省级科技计划项目58项。全市已有7个镇被列为省级专业镇技术创新试点。知识产权保护得到加强，申请专利879项，授权544项，居全省前列。市电子政务统一网络平台进一步完善，《中国瓷都——潮州信息网》已开通，山区信息化建设正加紧实施。科普教育取得新进展，全市已建立各级科普教育基地10个。

教育工作取得新成效。“普九”成果得到巩固和提高，素质教育稳步推进。大力发展普通高中教育，优质学位达78.2%。金山中学、高级中学新校区正加紧建设。对68所农村中小学校进行了调整，等级学校建设取得好成绩。老区山区小学改造第二批50所已经完成。积极推进新课程改革，加强中小学生思想道德建设。教学质量稳步提高，高考取得较好成绩。职业教育改革进一步深化。“校校通”建设加快，有54所乡镇中心小学已配备计算机教室。市教育信息网正式开通。成功承办了第九届全国“华罗庚金杯”少年数学邀请赛总决赛，我市代表队获得团体亚军。重视教育扶贫工作。有26349名农村贫困家庭子女获义务教育阶段免收书杂费优待，还有224名市直国企下岗特困职工子女得到资助。

卫生工作稳步推进。公共卫生体系建设步伐加快。市县疾控中心建设加紧推进。建成市、县、镇三级疫情直报网络。“120”医疗急救中心进一步完善。市中心医院内科大楼、传染病区正抓紧施工。农村卫生工作稳步推进，县级预防保健机构和镇级卫生院建设得到加强，改水改厕深入开展。新型农村合作医疗覆盖面逐步扩大，参加农村合作医疗人数86.7万人，普及率44.2%。

人口增长得到有效控制。健全“依法管理、优质服务、村（居）民自治、政策推动、综合治理”工作机制，确立“巩固一类，提高二类，消灭三类”的工作目标，大力推进计生工作重心下移，层层落实包干责任制，不断提高计生管理水平和服务水平。全市人口出生率9.98‰，自然增长率4.5‰，较好地完成了省下达的人口与计生工作任务。

环境与资源保护得到加强。认真抓好《潮州市生态建设规划》编制工作，推进“生态潮州”建设。韩江、枫江、黄冈河流域污染整治稳步推进。城市污水处理率达到60%以上。加强污染源的治理，推进重点污染源全面达标。建设项目全面落实环评制度，“三同时”执行率达到100%。在省组织的环保责任制考核中连续四年取得优秀等次。加大基本农田保护力度，坚决执行基本农田保护区“五个不准”规定，实现年度耕地占补平衡。认真开展土地市场治理整顿，清查处理各类违法用地行为。矿产资源管理进一步加强。

侨务工作取得新进展。加强对做好侨务工作的调查研究，积极开展对外联络宣传工作。组团赴香港、澳门和泰国、新加坡、马来西亚等地进行考察访问，进一步联络了乡谊，促进了合作。举行第二批荣誉市民授荣仪式，激发广大旅外乡亲爱国爱乡的热情。全市接受海外侨胞、港澳同胞捐资36宗，折合人民币7085.7万元，创近年新高。

体育、广播电视等各项事业全面进步。第五届体育节圆满结束。全民健身广场建成并向市民开放。我市输送国家集训队的女跳水队员林娜参加世界杯系列大赛共夺得金牌2枚，银牌和铜牌各1枚。少年围棋队参加全国“育苗杯”比赛获团体总分第一名。成功承办了省青少年武术散打锦标赛。广播电视宣传工作成绩显著。加强国防、民兵预备役和双拥工作，第五次获得省“双拥模范城”称号，被评为省“征兵工作全优单位”。巩固和扩大了殡葬改革成果。乡镇撤并工作圆满完成。人事、编制、物价、统计、人防、气象、地震、档案、市志、宗教等工作都取得新进展。

（八）人民生活水平不断提高，社会保障逐步完善

城乡居民收入有所增加。市区居民人均可支配收入8351元，增长7.6%。农民人均纯收入4123元，增长4.1%。居民消费层次不断提高，交通、通信工具及服务、文教娱乐服务消费支出有较大幅度增长。

就业和再就业工作稳步发展。认真落实再就业扶持政策和措施，加大就业岗位开发力度，建立健全劳动力市场，加强创业培训工作，推进城乡统筹就业。全市新增就业岗位2.6万个，下岗失业人员再就业10086人；城镇登记失业率2.64%，低于预期控制目标；培训各类技能人才5630人，培训农民工9591人，转移、输出农村富余劳动力22114人。有1119名农村贫困家庭应届初、高中毕业生免费接受技工教育。

社会保险覆盖面进一步扩大。医疗保险制度改革加快，社会保险基金实现较大增长。全市参加养老保险23.41万人、失业保险21.06万人、工伤保险13.95万人、医疗保险2.55万人，分别增长10.6%、11.2%、17.3%和21.6%。养老、失业、工伤、医疗四项保险基金收入34745万元，增长31.3%。支付各项保险待遇24882万元，实现基金收支平衡的目标。

社会福利事业不断进步。最低生活保障范围进一步扩大。全市列入低保4.12万人，同比增加6161人，低保面达到1.65%。城乡福利设施建设得到加强。市社会福利院动工建设，新扩建乡镇敬老院9所。全市“星光老年之家”累计41个。实施了新的《潮州市老年人优待办法》。老年大学挂牌开学。李嘉诚基金会发起的“关心是潮流”医疗扶贫项目启动仪式在我市举行。市慈善总会组织的“社会亲情关爱孤儿助学活动”、“健康直通车义医活动”、“资助贫困家庭大学生活动”等，受到广泛的好评。抓好残疾人陶瓷培训扶贫基地等载体建设，多形式开展残疾人就业培训。残疾人康复工作和文体活动也取得较好成绩。

住房解困工作扎实有效。积极筹措资金，购置、改造廉租房166套，解决了市城区166户双困户和危房户的住房难问题，提前一年实现“三年解困”目标。扎实抓好5个示范点的工作，逐步实施农村安居工程。

（九）社会经济秩序保持稳定，精神文明建设进一步加强

社会治安保持稳定。坚持“打防结合，预防为主”的方针，重拳打击“两抢两盗”等犯罪活动。积极推进“禁毒03工程”，坚决扫除“黄赌毒”等社会丑恶现象。进一步完善社会治安防控体系，加强流动人口和出租屋的管理。落实综合治理各项措施，认真排查化解社会矛盾，妥善处置群体性事件，营造良好的社会治安秩序。

市场经济秩序逐步规范。深入开展食品安全专项整治。实施药品放心工程。认真做好地方金融资产清收工作。全面开展打击制售假冒伪劣商品行为。继续打击走私和整顿税收秩序。加大知识产权保护和“扫黄打非”工作力度。加强安全生产工作，全面完成省下达的安全生产控制目标任务。市场经济秩序进一步好转。

精神文明建设有新进展。认真开展创建学习型领导班子、推动创建学习型社会的活动，不断推进理论教育和思想建设。加强“爱国、守法、诚信、知礼”现代公民教育，深入开展未成年人思想道德建设，提高全民思想道德素质。大力弘扬新时期潮州人精神，广泛开展文明城市、星级文明示范小区、文明社区、文明村镇、文明单位和文明窗口创建活动，提高了城乡文明程度。

（十）民主法制建设得到加强，效能建设取得显著成效

大力推进依法行政。坚持向市人大及其常委会报告工作、向政协通报情况的制度，自觉接受监督。全年承办人大议案、代表建议和政协委员提案159件，均在法定期限内办结。认真贯彻执行《行政许可法》。开展全员培训，提高行政执法水平。清理行政许可事项，认定行政许可实施机构减少为39个，行政许可事项减少为245项，清理涉及行政许可规范性文件的工作进展顺利。法制工作得到加强，制订实施规范性文件34个。“四五”普法扎实推进，法律援助工作取得新成绩。落实信访工作责任制，规范市长、县长接访日工作，广泛听取人民群众意见，解决了一批突出问题。

加强行政执法监察。开展征用农村集体土地和土地征用款专项执法监察，推进房屋拆迁管理规范化，切实解决拖欠工程款和民工工资问题。认真落实中小学收费“一费制”、高中入学“三限制”规定，严肃查处教育乱收费行为。进一步落实药品招标采购制度，纠正医药购销中的不正之风。加大对公路“三乱”的监督检查力度，实现全市公路基本无“三乱”目标。

深化机关效能建设。完善和落实机关内部的管理制度，推进政务公开工作，严格行政服务办理时限，扎实开展行政效能监察。“群众评机关”活动收到较好成效。贯彻执行党内监督条例，促进领导干部廉洁从政。落实党风廉政责任制，严格履行政府廉政“六项承诺”。制订实施有关规定，全方位加强市级财政投资项目的监管力度。成立市行政服务中心，进一步改善投资软环境。

各位代表：过去的一年，我们克服了各种困难，取得显著的成绩。这是省委、省政府和市委正确领导的结果，是全市人民勤劳拼搏的结果。这些成绩的取得，离不开市人大及其常委会和人民政协的监督支持，离不开驻潮部队、中央和省驻潮单位，各民主党派、人民团体和港澳台同胞、海外侨胞的鼎力支持。在这里，我代表市人民政府表示衷心的感谢和崇高的敬意！

我们也清醒地看到，我市经济和社会发展中仍然存在一些突出的困难和问题。主要是：经济基础仍较薄弱，产业竞争力还不够强，经济结构调整任务艰巨；能源、资金、企业用地紧缺，企业生产成本上升，在一定程度上制约着经济发展和效益提高；利用外资总量偏小，招商引资工作亟待进一步加强；农民增收难度较大，促进城镇就业和农村富余劳动力就业的任务仍然繁重；财政支出刚性增长，收支矛盾十分突出；政府机关作风建设存在薄弱环节，投资软环境有待进一步改善。对以上问题，市政府将采取切实有效的措施，认真加以解决。

2005年的工作意见

今年是全面实现“十五”计划目标、衔接“十一五”发展的重要一年，也是我市经济社会推进新发展、实现新跨越的关键一年。政府工作的总体要求是：以邓小平理论和“三个代表”重要思想为指导，进一步树立和落实科学发展观，认真贯彻党的十六大、十六届三中、四中全会和中央经济工作会议精神，认真贯彻胡锦涛总书记视察广东重要讲话精神，认真贯彻省委九届六次全会精神和省委、省政府的各项工作部署，认真贯彻市委十一届六次全体会议精神，以全面建设“活力潮州、文化潮州、生态潮州、法治潮州”为目标，抓住机遇，深化改革，扩大开放，加快发展，不断壮大经济规模，提高经济增长质量和效益；大力推进文化建设，加快社会事业发展，提高城市文明程度和公民道德文化素质；坚持依法治市、依法行政，加强民主法制建设，维护社会稳定；坚持以人为本，解决群众生产生活困难，提高人民生活水平，构筑和谐社会，为使潮州成为充满活力、富有魅力、文明和谐、富裕安康的广东“东大门”而努力。

贯彻落实上述总体要求，做好今年的工作，必须坚持“抓落实、重创新、促发展”的方针，做到“始终把握一个主题，紧紧抓住两个关键，努力促进三个有效增长，推进实施四项战略，做大做强五大经济”。

“始终把握一个主题”，就是以发展为第一要务，采取更加扎实有效的措施，创造性地开展工作，保持经济持续较快发展，促进社会全面进步，加快构筑和谐社会。

“紧紧抓住两个关键”：一是抓住调整优化产业结构这个关键。千方百计招商引资，做大做强支柱产业，大力发展高新技术产业、重化工业和环保产业，加快发展以旅游业为龙头的第三产业，提高产业竞争力。二是抓住转变经济增长方式这个关键。坚持以提高质量和效益为中心，把经济工作的重点转到提高经济增长质量和效益上来，构建节约型的产业结构和消费结构，努力建设节约型经济、节约型社会。

“努力促进三个有效增长”：一是努力促进固定资产投资有效增长。继续落实中央宏观调控政策，多渠道筹措建设资金，加快建设一批关系全局和长远发展的基础设施项目，不断改善经济发展环境。二是努力促进外贸出口有效增长。坚持科技兴贸，优化出口商品结构，提高出口商品质量，推动出口市场多元化。三是努力促进消费需求有效增长。不断增加城乡居民收入，改善消费环境，培育和扩大消费热点，促进住房、汽车、教育等消费健康发展。

“推进实施四项战略”：一是推进实施科技创新战略。完善以企业为主体的科技创新体系，鼓励和引导企业加大科技投入，增强自主研发能力，切实加强知识产权保护。二是推进实施品牌带动效应战略。继续开展争创品牌活动，努力扩大品牌综合效应，在全社会形成培育品牌、发展品牌、宣传品牌、保护品牌的良好氛围。三是推进实施市场多元化战略。坚持内外并重，积极开拓国内外市场，走市场多元化道路。四是推进实施人才兴市战略。努力营造培养、吸引、使用人才的良好环境，充分发挥人才效应，促进全市经济和社会的发展。

“做大做强五大经济”：一是做大做强民营经济。要进一步落实鼓励和支持民营经济发展的政策措施，引导民营企业推进体制创新、科技创新和管理创新，促进民营企业大发展大提高。二是做大做强县域经济。落实加快县域经济发展的决定及配套政策，以工业化、城镇化、农业产业化为主要途径，以发展民营经济和加大招商引资力度为主要措施，以培养一批专业镇和特色产业群为主要载体，促进县域经济更快更好地发展。三是做大做强特色经济。进一步打响“中国瓷都”和“中国婚纱晚礼服名城”等区域品牌，全力打造陶瓷产品研发中心、制造中心、集散中心和婚纱晚礼服设计中心、商贸中心、时尚发布中心。四是做大做强文化经济。进一步整合丰富文化资源，促进文化与经济的融合，全力打造“文化潮州”。五是做大做强港口经济。要加快港口经济区建设步伐，积极引进重化工业和环保产业，培育新的经济增长点，增强经济发展后劲。

2005年，我市经济社会发展的主要预期目标是：生产总值增长11%，社会消费品零售总额增长12%，全社会固定资产投资增长30%，外贸出口增长10%，地方财政一般预算收入增长11.5%，居民消费价格涨幅控制在2%左右，城镇登记失业率控制在3%以内，城镇居民人均可支配收入实际增长8%，农民人均纯收入实际增长4.5%，人口自然增长率控制在5.9‰以下。

为实现今年经济社会发展预期目标，必须做好以下工作：

（一）进一步调整优化产业结构，发展壮大特色经济

1、大力发展特色农业，提高农业经济效益

加强特色农业基地建设。在稳定粮食生产的同时，积极发展潮州名茶、生柑、花卉和深水网箱养殖等特色农业。以重塑“潮州柑”品牌为重点，建设生柑无病育苗基地，培植优质生柑品种，继续鼓励和扶持种柑大户。实施茶叶品牌战略，认真办好潮安凤凰山无公害优质茶、饶平田峰山有机茶生产基地。进一步提高加工水平，改善产品包装，推动潮州茶叶的品牌和质量标准建设。加强对花卉种植业的指导，稳定和扩大潮安县金石、龙湖两个花卉基地的规模。切实抓好柘林湾养殖布局调整，大力发展深水网箱养殖，建设新型环保网箱养殖示范区。积极推进优质鱼类、对虾出口创汇标准化养殖基地和鲍鱼、贝类等无公害养殖基地的建设。加快三百门国家一级渔港建设步伐和柘林国家一级渔港的规划申报工作。继续因地制宜发展“一乡一品”特色产业。切实抓好农田水利基本建设。

积极推进农业产业化。着力培育和壮大农业龙头企业，提高龙头企业带动农户增产增收的能力。进一步搞活农产品流通，大力扶持重点农产品流通企业，加快饶平水产品批发市场的建设。积极组织企业参加国家和省各种经贸活动，推介优势农产品。加强农业技术推广，创新农业技术推广体制，推动农业技术进村入户。建立完善农产品质量安全管理体系，逐步推行农产品市场准入制度。引导农民运用标准化技术发展农业生产。加强动植物疫情监测，完善疫情报告、预警机制。

努力拓宽农民增收渠道。全面贯彻落实中央一号文件和国家、省扶持“三农”的政策，切实增加农民收入。采取切实有效的措施，全面完成农村二轮土地承包工作，解决“两田分离”的遗留问题，确保承包土地面积、地块、承包合同、承包经营权证书“四到户”。认真做好免征农业税、粮食直接补贴、水稻良种补贴、农业机械化补贴等工作。办好农村改水改厕、道路硬底化、危房改造、新型合作医疗、税费改革等好事实事。完善村务公开和民主管理形式，加强农村集体经济审计监督。创新扶贫开发机制，切实帮助贫困村改善生产生活条件。加强对农民转移就业的技能培训和创业培训，促进农村劳动力转移。加快中心镇建设，完善城镇基础设施，增强城镇吸纳劳动力就业和发展第三产业的能力。

2、做大做强特色工业，提高产业竞争力

继续实施品牌战略。以荣获“中国瓷都”、“中国婚纱晚礼服名城”等称号作为新的起点，完善产业发展规划，打造陶瓷产品研发中心、制造中心、集散中心和婚纱晚礼服设计中心、商贸中心、时尚发布中心。继续培育更多的名牌产品和名牌企业，陶瓷、服装要在争创国家名牌产品方面实现新突破。

健全科技创新体系。加快建设信息化服务平台，进一步发挥“中国瓷都（潮州）信息网”的作用，依托市民营科技园网络中心，为企业提供网上技术咨询、技术展示、技术交易等服务。加强与高等院校及科研机构合作，提升现有企业工程技术研究开发中心的设备水平、技术力量和服务功能，增强创新能力。积极争取建立国家级日用陶瓷生产力促进中心、国家陶瓷产品质量监督检验中心和省食品质量安全监督检验中心。加快陶瓷职业技术学院建设，形成教研、实验和生产相结合的特色教学体系。

加快技术改造步伐。加大技改资金投入，实施一批符合产业政策、有较高技术含量的技术改造项目，鼓励引导符合条件的企业参与国家、省技术研发项目投标。积极申报国家和省2005年重点技术改造、技术创新项目计划。抓好30个重点技术改造项目，年度投资计划13亿元，促使项目尽快投产。加快新技术、新材料、新设备推广和运用，构建节约型产业。

创新企业管理体制。引导和鼓励企业创新体制，走集约化扩张的路子。扶持龙头骨干企业，积极稳妥推进股份制改造和改制上市。加快发展具有自主知识产权和著名品牌的大型企业集团，发展一批“强、精、专”的中小产业群。学习借鉴先进管理经验，提高企业家素质，加强内部管理，建立现代企业制度。

增强产业集散效应。积极组织、引导企业参加国内外的各类经贸活动，加强与各大连锁店的合作，在全国各大城市建立销售网络，争取陶瓷产品进入2008北京奥运市场。认真办好首届中国瓷都（潮州）——国际陶瓷博览会，为企业提供展销产品、引进科技成果和专业人才的最佳机会。

3、加快建设“文化潮州”，提高第三产业发展水平

推进古城文化旅游区建设。完成许驸马府维修工程，修复广济桥进入第二期施工，动工修复己略黄公祠和太平路牌坊。创办潮州民俗展览馆和广济桥史陈列馆。做好笔架山宋窑遗址修复和辟建潮州古陶瓷陈列馆的前期工作。积极组织一批文物申报国家、省级重点文物保护单位。进一步完善古城文化旅游区的配套设施。

振兴和发展工艺美术行业。重新组建潮州市工艺美术协会，成立潮州工艺美术研究院，设立专项保护资金，加强对濒危失传的特种工艺美术门类的抢救性保护。开展评选潮州工艺美术师活动，对有重大贡献的工艺美术人才授予潮州市工艺美术大师称号。对国家和省级工艺美术大师、高级工艺美术师给予政府津贴。制定扶持措施，引导企业开发有特色、易携带、可收藏的工艺品和旅游纪念品，促进工艺美术产品市场化、产业化。

加强旅游项目和配套设施建设。完成旅游总体规划修编工作。抓好韩江市区段和东岸片区的规划和开发，配套完善滨江长廊、人民广场、红山森林公园、西湖公园、慧如公园、淡浮院等景区景点，搞好绿岛山庄、龙湖寨、道韵楼、中华名茶园、白水岩风景区等五个省旅游扶贫项目的建设。加快凤凰山生态旅游区、星湖温泉度假村的建设步伐。

加大旅游宣传促销力度。精心包装和大力推介具有特色的旅游线路，加强与粤东旅游圈、闽粤赣十三地市，以及泛珠三角的旅游合作。办好第二届潮州文化旅游节，在澳门召开第十三届国际潮团联谊会期间举办潮州美食文化节，充分展示潮州的文化底蕴、旅游魅力和发展环境。

加快文化设施和文化产业发展。积极实施潮州市文化建设发展纲要。扎实抓好文化艺术馆、博物馆、图书馆、档案馆、饶宗颐学术馆等文化项目的前期工作，确保今年动工建设。加强与中山大学等院校的合作，弘扬潮州文化。加强基层文化站建设，开展广场、社区、校园、企业文化活动，营造城乡文化氛围，增强名城文化魅力。积极发展文化产业，在发展印刷、光盘等文化产业的同时，高度重视和培育新兴文化产业。扶持发展“宇航鼠”动漫产业项目，打造动漫文化品牌，带动相关产业发展。

大力发展现代服务业。扩大服务业对外开放，壮大物流、信息、咨询等现代服务业，改造提升商贸、餐饮等传统服务业，发展社区服务和中介服务，推动第三产业向多元化、高级化发展。增加城乡居民收入，优化消费环境，拉动消费需求。培育和拓展汽车、住房、教育、娱乐、体育和健身等消费热点，提升消费结构。

（二）进一步提高对外开放水平，增强经济整体实力

提高利用外资水平。认真贯彻落实《关于加快利用外资的若干意见》、《潮州市招商引资奖励暂行办法》，加强对外来投资者的宣传，调动全社会参与招商引资的积极性。更加重视侨务工作，加强与世界各地华侨华人的联系与合作。继续实行领导挂钩重点项目制度，对现有外商投资企业要加强服务，鼓励其增资扩产。对在谈的引资项目，尤其是首届文化旅游节签约项目要抓好跟踪落实。发挥侨乡优势，创新引资方式，实行“走出去”招商和“引进来”招商相结合、园区招商和企业招商相结合、主动招商和以商引商相结合、自行招商和委托招商相结合、传统招商与网上招商相结合等多种招商方式。抓住世界新一轮产业转移的机遇，加大对台湾地区和日、韩及欧美各国的招商引资力度。抓住泛珠三角区域经济合作全面启动的有利时机，积极吸纳国内投资，主动承接珠三角产业转移，动员潮籍在外地投资的企业家回家乡投资。搞好开发区和工业园区等招商引资载体建设。完成潮州港规划编制工作，加快建设步伐，大力发展能源工业、重化工业和环保产业，推进“以港兴市”战略的实施。进一步搞好市行政服务中心的建设，为投资者和企业提供简便快捷、优质高效的服务。重视和解决企业反映的有关问题，切实维护投资者的合法权益。

提高外贸出口竞争力。推动贸易方式和市场多元化，巩固港澳、欧美日、东盟等传统市场，大力开拓非洲、南美、俄罗斯等新兴市场。实施科技兴贸，坚持以质取胜，优化出口商品结构，提高出口商品附加值。在扩大陶瓷、服装、食品、不锈钢等产品出口的同时，进一步提高机电产品、高新技术产品出口比重。加强应对技术壁垒及反倾销等有关知识的培训和宣传，健全贸易摩擦预警机制，引导企业增强风险意识，提高应对国际贸易摩擦的能力。进一步落实出口退税政策。加强与口岸查验部门的协调沟通，优化通关环境，完成车检场搬迁工作。

（三）进一步加快基础设施建设，不断改善经济和社会发展环境

认真执行国家各项宏观调控政策。积极拓宽融资渠道，继续抓好一批关系全局和长远发展的城市和基础设施建设，更好地发挥投资需求对经济的拉动作用，不断改善经济和社会发展环境，提升城市竞争力。

水利建设方面：积极配合加快建设潮州供水枢纽，争取将饶平县列入供水调配范围。认真抓好城乡水利防灾减灾工程和小型水库除险加固等工程。加紧做好韩江南堤护岸加固的前期工作，跟踪落实好潮安县内洋排涝整治工程立项工作，争取省的资金支持。

能源建设方面：继续配合搞好三百门电厂一期工程建设，抓紧做好二期工程的前期工作。加快城乡电网改造和输变电工程，以及凤凰水电厂二期更新改造工程建设。抓好500千伏潮州输变电项目的前期工作。

交通建设方面：配合做好厦深铁路潮州段前期工作，争取省高速公路网规划“漳州—玉林”高速公路潮州段、S232线护堤路段提前开展前期工作，加快韩江北桥、潮揭高速公路、镇通村公路硬底化建设，确保完成S334丰柏线工程、县通镇三级公路工程建设任务。动工改造S335线潮州段（东山路口至深坑桥）和建设中心客运站。

城市建设方面：认真做好城镇体系规划和城市总体规划的修编工作。扎实推进创建“国家园林城市”活动。动工建设绿榕西、南路，改造枫春路、潮枫路、中山路、北站二路。搞好城市供水设施配套建设。逐步治理城市排水系统。加快市垃圾卫生填埋场和陶瓷废物处理中心建设。进一步落实城市管理长效机制，提高城市管理水平。

（四）进一步发展各项社会事业，促进经济社会可持续发展

推进精神文明建设。深入开展“爱国、守法、诚信、知礼”现代公民教育活动，提高公民素质；采取学校、家庭、社会结合的方式，以“守法、诚信、知礼、尊重”教育为内容，加强未成年人思想道德建设；开展富有潮州特色的群众性精神文明创建活动，努力增强创建实效，增进人文关怀，促进社会和谐。

加快实施“人才兴市”战略。推进专业技术人员继续教育培训，不断提高各类人才的创新能力。深化职称制度改革，建立科学准确的专业人才评价机制，加大为民营经济和农村乡土人才服务的力度。加强人才市场建设，提高人才资源市场化配置服务水平。落实国家和省关于人才工作的各项政策，努力营造培养、吸引、使用人才的良好环境。

增强科技创新能力。认真组织申报、实施国家和省重点科技计划项目，大力发展国家级、省级高新技术企业，加快科技攻关和技术成果的转化。力争科技进步贡献率达到44.5%，高新技术产品产值达到82亿元。认真落实扶持民营科技园建设的政策措施，鼓励民科企业发展高新技术项目。建设科技创新创业综合服务中心，搞好市级技术创新服务中心试点。加快信息化建设，加强电子政务规范化管理，做好市政府公众信息网的更新维护。

促进教育事业协调发展。完善“以县为主”的农村教育管理体制，确保农村义务教育经费不低于税费改革前的水平。巩固和提高“普九”成果，继续调整学校布局，促进农村教育均衡发展。大力发展普通高中和中等职业教育，加快金山中学、高级中学、卫生学校新校区建设和技工学校扩建工程。推进新课程改革，加快信息化建设，提高办学质量。深化办学体制改革，扶持和鼓励民办教育发展，规范各类教育办学行为。落实“减、免、缓、助”等教育扶贫政策措施，帮助经济困难学生完成学业。努力完成第三批32所老区学校的改造工程。重视外来工子女入学问题。

提高公共卫生保障水平。加快市中心医院内科大楼、潮安县人民医院、市、县疾控中心和市、县重点医院传染病区建设。动工建设妇女儿童医院，配套完善市“120”医疗急救中心。完善突发公共卫生事件应急预案，建立健全应急救治队伍，提高防护水平和应对能力。严格疫情报告制度，防治各类传染疾病。深入开展爱国卫生运动，巩固和提高创建省卫生城市成果。积极实施农村新型合作医疗制度，落实市、县、镇三级财政配套资金，力争新型农村合作医疗普及率达到55%。

统筹体育、广播电视等各项事业发展。认真备战省运会，办好第十三届市运会和第六届体育节。承办好2005年广东省青少年围棋锦标赛。搞好国民体质监测工作，完善全民健身广场的设施。实现广播电视网络市、县、镇三级联网，推进有线数字电视整体转换，提高广播电视传播的服务水平和影响力。继续搞好经济普查工作。加强全民国防教育，搞好民兵预备役建设，积极筹划创建全国双拥模范城。进一步做好统计、人防、气象、地震、档案、地方志等工作。此外，要抓紧做好“十一五”规划的制订工作，提高规划的预见性、科学性和指导性。

坚决控制人口过快增长。进一步落实人口与计划生育目标管理责任制，加强计生队伍和服务网络建设。不断完善以农村计划生育家庭奖励制度为主要内容的计划生育利益导向机制。要在抓好经常性工作的基础上，适时组织计生集中服务活动，促进计划生育政策的全面落实。采取综合治理措施，遏制出生性别比异常升高现象。加大依法征收社会抚养费的工作力度。

合理利用土地和海洋资源。认真抓好市、县、镇土地利用总体规划的修编工作。落实耕地保护目标责任制，严格保护基本农田，深入整治土地市场秩序。坚持依法、合理利用土地，坚决制止和纠正各类违法用地行为，尤其要对违法违规圈占、出租土地进行清理。抓好农民征地补偿款的清欠工作。坚持依法治矿，严肃查处乱采滥挖、破坏矿产资源的行为。做好矿产资源规划编制、地质灾害防治、地质遗迹管护工作。加大海域综合开发和管理力度，坚决制止利用无度、开发无序、使用无偿的用海行为。

推进“生态潮州”建设。做好生态保护工作，完成《潮州市生态建设规划》。积极创建生态园、生态村、生态镇和自然保护区。抓好韩江水源涵养林等重点生态工程建设，建立森林资源与生态状况综合监测体系，努力提高森林生态体系质量。以环保责任考核为中心，以实施治污保洁为重点，抓好污染控制、生态保护、辐射环境管理。继续实施韩江、枫江和黄冈河流域污染整治工程。抓好潮安、饶平两县生活污水和垃圾处理项目的规划建设。严格执行《环境影响评价法》，加强建设项目环境管理。引导和帮助企业做好清洁生产和环境标志的认证工作。狠抓重点工业污染源全面达标工作，确保年底前顺利通过省的验收。

（五）进一步实施民心工程，切实改善群众生产生活条件

加强就业和再就业工作。强化就业再就业目标责任制考核。加大就业岗位开发力度，全年新增就业岗位2.8万个。建立健全覆盖城乡劳动力市场信息网络，完善公共就业服务体系。落实小额担保贷款、社保补贴和岗位补贴等再就业扶持政策，大力实施“零就业家庭”就业援助制度，开展农村贫困户就业扶贫行动，促进下岗失业人员等困难群体就业再就业。加强职业技能培训，加快各类技能人才培养。强化劳动保障监察和劳动关系协调，维护劳动者的合法权益。

强化社会保险扩面征缴。继续扩大社会保险覆盖面，提高非公有制企业的参保率。进一步完善养老保险、失业保险、医疗保险和工伤保险。强化社保基金征缴和监管工作，防止挤占、挪用和欺骗冒领社会保险金的行为。

抓好农村饮水工程建设。加快建设饶平沿海片供水工程，确保下半年供水到各镇。扩建桥东水厂，让意溪、磷溪、官塘、铁铺等地的群众饮上洁净的自来水。抓好潮安县韩西、韩东片供水工程建设。积极筹措资金，年底前解决14.8万农村人口饮水特别困难的问题。

推进安居工程建设。全面排查市城区危房户基本情况，按照实际情况分清先后缓急，切实做好退迁安置工作。加快农村安居工程示范点建设，千方百计筹措资金，逐步推进农村安居工程。

完善社会救助体系。进一步做好城乡居民最低生活保障扩面工作，妥善解决农村五保户和特困户的生活困难。抓好救灾救济工作，安排好受灾群众生活。发展社会福利和残疾人事业，推进“千间敬老福星工程”活动，加快市福利院建设，实施“南粤助残工程”。加强老龄工作。广泛开展社会慈善活动。重视解决优抚对象、企业军转干部以及特殊群众生活保障问题。积极开展法律援助工作，维护弱势群体的合法权益。

（六）进一步转变政府职能，服务经济建设和社会发展

深化各项改革，增强发展动力。以产权制度改革为核心，完善国有资产管理体制和监管方式。加快推行公益性项目代理建设制，引入竞争机制，鼓励社会资本进入垄断行业。继续落实扶持民营经济发展的各项政策。加快发展土地、技术、劳动力等要素市场。规范有形建筑市场和房地产市场交易行为。积极培育发展各类中介组织、行业协会。深化农村信用社改革，加强和改善农村金融服务。

加强市场监管，整治经济秩序。继续抓住关系人民群众身体健康、生产安全的突出问题，深入开展食品药品生产流通专项整治，重点查处生猪黑屠私宰行为。严厉打击制假售假行为，加强专利和保护知识产权工作。扎实开展反走私联合行动和专项斗争。加强“扫黄打非”和网吧管理，净化文化市场。抓好在建工程的监督检查，规范商品房销售行为。继续推进信用等级分类管理，认真办好潮州信用网。

加强监督管理，搞好安全生产。层层落实安全生产责任制和责任追究制，开展经常性的安全执法检查，抓好安全生产专项整治。加强燃气市场的管理和特种设备的现场安全监察。大力整治交通秩序和运输市场秩序，继续开展清查无牌无证、报废和非法拼装机动车工作，重视北部山区交通安全管理。抓好非公有制企业安全监管试点工作。推进消防工作社会化，在重点乡镇和企业组建消防队。

加强社会管理，整治治安环境。把维护社会稳定摆在各项工作的首位，妥善处理群体性事件。严厉打击黑恶势力犯罪、严重暴力犯罪、毒品犯罪以及“两抢两盗”等多发性侵财型犯罪。深入开展打击“六合彩”和禁毒等专项斗争。改进对流动人口和出租屋的管理。落实“网格化”巡逻执勤。完善以110为龙头、以巡警为依托、多警种联合的快速反应机制。深入开展普法教育，增强全民法制观念。构建群防群治网络，增强基层民事调解和民间纠纷调处的能力，创造和谐稳定的社会环境。

加强财税征管，确保重点支出。加强税收征管，依法组织收入，加大稽查力度，严厉打击各种偷逃税行为。努力拓宽非税收入渠道，规范非税收入管理，对部门预算外收入实行收支脱钩和收缴分离。进一步调整优化支出结构，优先保证财政供养人员的工资发放和农业、教育、科技等重点支出。严格控制一般性支出，严禁铺张浪费。充分运用法律手段，抓好金融债务的追收和清偿工作。继续搞好国库集中支付试点工作，扩大部门预算改革的范围。稳步推进财政支出绩效评价工作。继续完善政府采购各项规章制度，扩大政府采购的范围。进一步完善财政监督机制。

加强公共服务，建设法治政府。自觉接受人大和政协的监督，坚决执行市人大及其常委会的各项决议，认真办理人大代表的建议和政协委员的提案。对牵涉面广、群众关注的重大事项，实行社会公示制度和社会听证制度。继续通过“市长电子信箱”、“市长接访日”等形式，广泛听取群众意见。坚持依法行政，全面推行行政执法责任制、执法公示制。深入贯彻实施《行政许可法》和《全面推进依法行政实施纲要》，简化和规范行政许可程序，强化对行政许可行为的监督。加强机关效能建设，集中精力抓服务、促发展。积极探索推行政府法律顾问制度。深入开展保持共产党员先进性教育活动和“理想、责任、能力、形象”教育，完善公务员选人用人机制，提高公务员队伍素质。推进政务公开，提高政务行为的透明度和公众参与度。严格执行廉洁自律各项规定，深入推进行风建设。加强对财政预决算、预算执行单位财务收支、专项财政资金、国有资产、政府投资建设项目的审计监督。进一步完善经济责任审计制度。强化行政监察工作，严肃查处违法违纪案件。重视基层政权建设，完成村（居）委换届选举工作。

各位代表，今年我们面临新的机遇，也会遇到新的挑战。我们要团结一致，奋力拼搏，努力推动全市经济和社会全面、协调、可持续发展，为建设充满活力、富有魅力、文明和谐、富裕安康的广东“东大门”而奋斗！
